# Serotonin syndrome associated with concomitant tramadol and linezolid therapy: a case report and literature review

**DOI:** 10.3389/fphar.2026.1825964

**Published:** 2026-05-29

**Authors:** Huixin Zhao, Kun Liu, Yuehong Zheng, Leng Ni

**Affiliations:** 1 Department of Pharmacy, North China University of Science and Technology Afffliated Hospital, Tangshan, China; 2 Department of Vascular Surgery, Peking Union Medical College Hospital, Chinese Academy of Medical Science and Peking Union Medical College, Beijing, China

**Keywords:** drug interaction, linezolid, pharmacovigilance, serotonin syndrome, tramadol

## Abstract

Serotonin syndrome (SS) is a potentially life-threatening adverse drug reaction resulting from excessive serotonergic activity in the central and peripheral nervous systems. Tramadol produces analgesia through μ-opioid receptor agonism while also inhibiting the reuptake of serotonin and norepinephrine. Linezolid an oxazolidinone antibacterial agent, possesses reversible, non-selective monoamine oxidase inhibitory activity. Through distinct pharmacological mechanisms, both agents enhance serotonergic neurotransmission and may theoretically increase the risk of SS when used concomitantly. We report the case of an elderly woman who developed serotonin syndrome shortly after receiving tramadol and linezolid for ischemic lower-limb pain complicated by severe infection. The patient fulfilled the Hunter diagnostic criteria, presenting with hyperthermia, tremor, myoclonus, hyperreflexia, muscle rigidity, diaphoresis, and flushing. The symptoms resolved rapidly after discontinuation of the suspected medications and initiation of supportive treatment. Although several real-world studies have suggested that the overall incidence of SS associated with concomitant linezolid and serotonergic agents is relatively low, this report highlights that individualized risk assessment remains essential in elderly patients with multiple comorbidities and polypharmacy. Careful evaluation of potential drug interactions and close clinical monitoring are therefore warranted when anti-infective and analgesic therapies with serotonergic properties are administered concurrently.

## 1 Introduction

SS is a potentially life-threatening drug-induced condition caused by excessive serotonergic activity in the central and peripheral nervous systems, primarily associated with overactivation of the 5-HT2A receptor (Bai et al., 2022). Clinical manifestations are highly variable, ranging from mild neuromuscular excitation to life-threatening hyperthermia and circulatory collapse (Bai et al., 2022). Numerous medications can induce SS by enhancing serotonergic neurotransmission through different mechanisms, among which antidepressants, monoamine oxidase inhibitors (MAOIs), and certain analgesics with serotonergic properties are the most frequently implicated agents (Mikkelsen et al., 2023).

Linezolid, an oxazolidinone antibacterial agent, exhibits reversible, non-selective MAOI-like activity in addition to its antimicrobial effects (Wali, 2025). Tramadol acts as a μ-opioid receptor agonist while also inhibiting the reuptake of serotonin and norepinephrine, thereby enhancing central serotonergic neurotransmission (Ates et al., 2025). In patients with severe infections, effective analgesia and potent antimicrobial therapy are often required simultaneously, making their concomitant use clinically plausible. Theoretically, the distinct effects of these two agents on serotonin metabolism and reuptake may produce pharmacological synergy, potentially increasing the risk of SS.

However, several recent real-world studies have suggested that SS is extremely rare among hospitalized patients receiving linezolid with or without concomitant serotonergic medications, even among those exposed to multiple or high-dose serotonergic agents (Kufel et al., 2023; Mitwally et al., 2022). Most previous studies have relied on population-based analyses, which may underestimate individualized risk in elderly patients with multiple comorbidities and polypharmacy. Here, we report a case of SS that developed after the addition of linezolid to a tramadol regimen in an elderly patient. Through analysis of this case and a review of the literature, we aim to discuss the diagnostic features, potential mechanisms, and clinical management strategies of SS, thereby providing insights for rational drug use and risk stratification in clinical practice.

## 2 Case presentation

### 2.1 Patient background and admission status

The patient was an 87-year-old East Asian woman (height 150 cm, weight 50 kg) who was admitted on 25 July 2025, with a 2-month history of intermittent claudication of the left lower extremity and a 1-month history of toe gangrene. On admission, vital signs were stable: temperature 36.5 °C, heart rate 61 beats/min, respiratory rate 19 breaths/min, blood pressure 105/55 mmHg, and SpO_2_ 98%. Physical examination revealed black necrosis of the third and fourth toes of the left foot, with markedly decreased local skin temperature and minimal exudate. Distal arterial pulses in both lower extremities were weak. Computed tomography angiography (CTA) of the lower extremities demonstrated occlusion of the left external iliac artery, bilateral femoral arteries, the left popliteal artery, and bilateral anterior and posterior tibial arteries. The patient had a medical history of hypertension, type 2 diabetes mellitus, and coronary artery disease. She denied any history of psychiatric disorders or long-term use of antidepressants, sedatives, hypnotics, or other central nervous system medications. No history of drug or food allergies was reported. Admission diagnoses included: (1) Fontaine stage IV atherosclerotic occlusive disease of both lower extremities; (2) coronary atherosclerotic heart disease; (3) grade 3 hypertension (very high risk); and (4) type two diabetes mellitus.

### 2.2 Initial treatment and laboratory findings

After admission, intravenous tramadol (100 mg once or twice daily) was administered for ischemic pain control. Of note, the patient had tolerated tramadol well previously, without any neuropsychiatric or neuromuscular adverse effects.

Laboratory tests on July 26 revealed a marked inflammatory response, with high-sensitivity C-reactive protein (hsCRP) of 134.39 mg/L and N-terminal pro-B-type natriuretic peptide (NT-proBNP) of 3,973 pg/mL. Empirical antimicrobial therapy was initiated. On July 30, laboratory results showed leukocytosis and further elevation of NT-proBNP (WBC 13.15 × 10^9^/L, hsCRP 135.76 mg/L, NT-proBNP >35,000 pg/mL). Intravenous imipenem/cilastatin (500 mg every 8 h) was initiated for antimicrobial treatment.

### 2.3 Clinical onset of serotonin syndrome after linezolid initiation

On July 31, wound culture results identified *Enterococcus faecalis* and Trichosporon asahii, and intravenous linezolid (600 mg every 12 h) was added. Within 5 h of linezolid initiation, the patient developed tremors involving the limbs, progressing to head tremor, accompanied by a low-grade fever (maximum temperature 37.9 °C). On August 1, body temperature increased to 38.7 °C, accompanied by persistent bilateral upper-limb tremors, myoclonus, hyperreflexia, muscle rigidity, profuse sweating, and facial flushing. The patient remained conscious and oriented throughout this period. Notably, inflammatory markers showed a declining trend (hsCRP 72.20 mg/L), and no electrolyte abnormalities or acute cerebrovascular events were identified. Creatine kinase was mildly elevated (239 U/L), accompanied by increased myoglobin (422 μg/L).

### 2.4 Diagnosis, management, and clinical course

**FIGURE 1:**
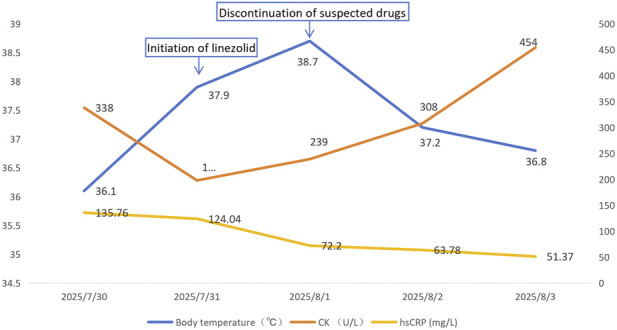
Temporal trends in body temperature and laboratory parameters associated with serotonin syndrome.

Based on the clinical manifestations and medication history, SS was strongly suspected. The diagnosis was subsequently confirmed according to the Hunter Serotonin Toxicity Criteria (Hölle et al., 2023). To further evaluate the causal relationship between the suspected medications and the observed adverse reaction, the Naranjo Adverse Drug Reaction Probability Scale was applied, yielding a total score of 7, indicating a probable adverse drug reaction (Manjhi et al., 2024). These findings suggest that the condition was likely associated with the concomitant use of tramadol and linezolid. Both medications were immediately discontinued. The patient’s vital signs and neurological status were closely monitored, with serial assessments of creatine kinase, blood gas parameters, and lactate levels. Supportive management, including aggressive fluid resuscitation, external cooling, and intravenous diazepam for tremor control, was initiated (Sweileh, 2024). Approximately 8 h after discontinuation of the suspected medications, the patient’s tremors and neurological symptoms began to improve. No new neurological abnormalities developed, and consciousness and orientation returned to normal within 48 h. Subsequent laboratory monitoring showed a gradual decline in inflammatory markers. Creatine kinase remained mildly elevated (454 U/L) but without further progression. The trends in body temperature and serotonin syndrome–related indicators are shown in Figure 1. Due to the patient’s poor overall condition, the family requested transfer to another hospital for further treatment, and long-term follow-up data were unavailable.

## 3 Discussion

### 3.1 Causality assessment of SS induced by concomitant linezolid and tramadol

In the present case, the patient developed high fever, tremor, increased muscle tone, and autonomic symptoms within 5 h after linezolid was added to an ongoing tramadol regimen. Notably, the patient had previously used tramadol regularly for analgesia without any neuropsychiatric or neuromuscular abnormalities. The onset of symptoms showed a clear temporal association with the initiation of combination therapy. After discontinuation of both linezolid and tramadol, along with supportive treatment, the patient’s symptoms gradually resolved, further supporting a drug-related adverse reaction. To objectively assess causality, the Naranjo Adverse Drug Reaction Probability Scale was applied (Manjhi et al., 2024), yielding a total score of 7 (Table 1). Taken together, the temporal relationship and positive dechallenge response support a probable association between SS and the concomitant use of linezolid and tramadol.

**TABLE 1:** Naranjo Adverse Drug Reaction Probability Scale for serotonin syndrome associated with Linezolid and Tramadol (score = 7, probable ADR).

Question	Score	Rationale
Are there previous conclusive reports on this reaction?	+1	Previous reports have described serotonin syndrome induced by tramadol or linezolid alone or in combination
Did the adverse event appear after the suspected drug was administered?	+2	The patient developed fever, tremor, and muscle rigidity shortly after linezolid was added to stable tramadol therapy.
Did the adverse reaction improve after the drug was discontinued or a specific antagonist was administered?	+1	Clinical symptoms gradually resolved after discontinuation of tramadol and linezolid
Did the adverse reaction reappear upon re-administration of the drug?	0	Re-exposure to the suspected drugs was not performed
Are there alternative causes that could have caused the reaction?	+2	No other plausible causes were identified
Did the reaction reappear when a placebo was given?	0	Placebo challenge was not performed in clinical practice
Was the drug detected in blood or other fluids at toxic concentrations?	0	Serum concentrations of tramadol and linezolid were not measured.
Was the reaction more severe when the dose was increased or less severe when the dose was decreased?	0	No clear dose–response relationship was observed
Did the patient have a similar reaction to the same or similar drugs previously?	0	The patient had no previous history of serotonin syndrome
Was the adverse event confirmed by any objective evidence?	+1	The patient exhibited typical clinical signs (myoclonus, hyperreflexia, muscle rigidity), elevated CK levels, and fulfilled hunter/Sternbach diagnostic criteria

### 3.2 Diagnosis and clinical manifestations of SS

SS is a potentially life-threatening adverse drug reaction caused by excessive serotonergic activity in the central and peripheral nervous systems (Maitland and Baker, 2022). The diagnosis of SS is primarily clinical, based on characteristic neuromuscular findings, autonomic instability, and a compatible medication history, as no specific laboratory biomarkers are available. The Hunter Serotonin Toxicity Criteria are considered the most accurate and clinically useful diagnostic tool, particularly in moderate to severe cases (Table 2) (Mikkelsen et al., 2023; Isbister et al., 2007).

**TABLE 2:** Hunter and Sternbach criteria for serotonin syndrome.

Criteria set	Diagnostic features (≥3 required for Sternbach; ≥1 for Hunter)
Sternbach criteria	Mental status changes, agitation, myoclonus, hyper-reflexia, diaphoresis, shivering, tremor, diarrhoea, incoordination, fever
Hunter criteria	Spontaneous clonus; inducible clonus + agitation or diaphoresis; ocular clonus + agitation or diaphoresis; tremor + hyper-reflexia; hypertonia + temperature >38 °C + ocular or inducible clonus

In the present case, the patient developed tremor, myoclonus, and hyperreflexia shortly after exposure to two serotonergic agents, accompanied by fever and profuse sweating. These manifestations fulfilled the Hunter diagnostic criteria, specifically “tremor with hyperreflexia” and “clonus with autonomic instability.” Meanwhile, inflammatory markers showed a declining trend compared with previous measurements, suggesting no evidence of worsening infection. Imaging and electrolyte evaluations also failed to identify alternative explanations for the neurological manifestations. Following discontinuation of the suspected medications, the patient’s symptoms improved rapidly, supporting the diagnosis of SS.

### 3.3 Pathogenesis of SS

SS is a toxic reaction caused by excessive serotonergic activity resulting from increased serotonin levels or impaired serotonin metabolism, typically following exposure to serotonergic medications (Wali, 2025). Consistent with this mechanism, SS in the present case developed after the combined use of linezolid and tramadol. The pathophysiology of SS is primarily attributed to excessive activation of 5-HT1A and 5-HT2A receptors, leading to neuromuscular hyperactivity, autonomic instability, and altered mental status (Badar, 2024; Kanova and Kohout, 2021). The synergistic overactivation of these receptor pathways constitutes the pathological basis of the clinical spectrum of SS, with disease severity influenced by the magnitude of 5-HT elevation, receptor sensitivity, and individual metabolic capacity.

In this case, the combination of linezolid and tramadol likely contributed to excessive serotonergic activity through complementary mechanisms. Linezolid has reversible, non-selective MAOI-like activity, which reduces serotonin metabolism and increases synaptic serotonin levels (Hasani et al., 2019). Tramadol inhibits serotonin and norepinephrine reuptake and may also enhance serotonin release (Ali et al., 2023). By simultaneously affecting two key pathways—metabolic inhibition and reuptake inhibition with release enhancement—these agents can produce additive serotonergic effects at the synaptic level, substantially amplifying serotonergic neurotransmission. This multi-target pharmacological interaction provides a mechanistic basis for SS induced by combination therapy.

However, despite the clear mechanistic plausibility, clinical evidence remains somewhat inconsistent. On the one hand, systematic reviews and meta-analyses suggest that the combination of linezolid with serotonergic medications may increase the risk of SS (SanFilippo et al., 2023). Analyses of pharmacovigilance databases have also classified combinations of linezolid with certain antidepressants and opioids as higher-risk regimens, recommending enhanced risk monitoring (Gatti et al., 2021). On the other hand, several real-world studies report a relatively low incidence of SS associated with these combinations, even questioning the strength of regulatory warnings regarding these drug interactions (Kufel et al., 2023; Mitwally et al., 2022; Karkow et al., 2017; Traver et al., 2022).

These discrepancies suggest that the clinical risk of serotonergic drug interactions is not constant but highly dependent on multiple factors—including patient characteristics, underlying disease status, number of concomitant medications, and drug exposure intensity. Mechanistic plausibility does not necessarily translate into a high population-level incidence; however, severe toxicity may still occur in susceptible individuals. The present case, involving an elderly patient with multiple comorbidities and polypharmacy, highlights the importance of individualized risk assessment in clinical decision-making. This case provides additional insight beyond population-based studies, as severe SS may still occur rapidly in vulnerable high-risk individuals despite the relatively low reported incidence of this drug combination.

### 3.4 Risk stratification for SS

Recent real-world studies have provided important insights into risk stratification for SS. At the drug level, selective serotonin reuptake inhibitors (SSRIs) are most strongly associated with SS and represent the drug class with the highest reported risk, followed by opioid analgesics and other antidepressants with serotonergic activity, such as those that inhibit 5-HT reuptake or promote its release (Gatti et al., 2021; Elli et al., 2024). This pattern suggests that medications directly increasing synaptic 5-HT concentrations play a central role in the development of SS. In line with this, tramadol inhibits serotonin reuptake, while linezolid exerts MAOI-like activity, leading to increased synaptic serotonin levels. Their concomitant use may produce additive serotonergic effects and an increased risk of SS.

At the population level, epidemiological studies suggest that SS risk is not evenly distributed among patients receiving serotonergic medications. Elderly patients have been reported to have a higher incidence of SS, and comorbid conditions such as renal disease and cardiovascular disease may further increase susceptibility (Elli et al., 2024; Di Salvo et al., 2024). In addition, concomitant use of multiple serotonergic agents appears to be a more important determinant of risk than the dose of a single drug (Di Salvo et al., 2024). In the present case, the patient was an 87-year-old woman with multiple comorbidities, including hypertension, diabetes mellitus, and coronary artery disease, and was exposed to two serotonergic agents (tramadol and linezolid). These factors may have collectively contributed to an increased susceptibility to SS.

In addition, pharmacogenetic variability in drug-metabolizing enzymes such as CYP2D6 and CYP2C19 has been suggested to contribute to inter-individual differences in susceptibility to SS (Milosavljevic et al., 2021; Poian and Chiavegatto, 2023). However, no genetic testing was performed in this patient; therefore, the role of pharmacogenetic factors in this case remains speculative.

Taken together, the occurrence of SS is not the result of a single pharmacological trigger but rather reflects a complex interaction among drug mechanisms, underlying disease status, concomitant medication use, and genetic background. Therefore, individualized risk assessment is particularly important in elderly patients, those with multiple comorbidities, and those receiving polypharmacy, as early identification of high-risk individuals may help reduce the occurrence of severe serotonergic toxicity.

### 3.5 Treatment principles for SS

The cornerstone of SS management is the immediate discontinuation of all suspected causative agents, followed by severity-based supportive care and symptomatic treatment, with the addition of 5-HT receptor antagonists when indicated (Wali, 2025; Isbister and Buckley, 2005). The onset of SS may occur within hours to several weeks after exposure to serotonergic agents, and clinical manifestations vary widely in severity. In severe cases, complications such as rhabdomyolysis, circulatory collapse, and other life-threatening conditions may develop; therefore, early recognition and prompt intervention are essential for improving patient outcomes.

Most mild to moderate cases have a favorable prognosis following timely drug discontinuation and supportive management. The fundamental therapeutic measure is withdrawal of all serotonergic medications, after which approximately 70% of patients experience gradual symptom resolution within 24–36 h (Kanova and Kohout, 2021). The clinical course in the present case was consistent with this pattern, as symptoms began to improve approximately 8 h after drug discontinuation and resolved completely within 48 h, supporting the importance of early withdrawal of serotonergic agents in the management of mild to moderate SS. For moderate cases, the serotonin receptor antagonist cyproheptadine is commonly recommended (Mikkelsen et al., 2023). The typical initial dose is 12 mg, followed by incremental doses of 2 mg every 2 h if symptoms persist, with a maximum of 32 mg within 24 h. Cyproheptadine has been shown to improve clonus, muscle rigidity, autonomic instability, and altered mental status, and is currently considered the most frequently used specific antagonist for SS (Petroianu, 2022; Deardorff et al., 2016).

Severe cases are often characterized by hyperthermia exceeding 41 °C and other life-threatening complications, requiring immediate intensive care management. Treatment typically involves sedation, administration of non-depolarizing neuromuscular blocking agents, endotracheal intubation, mechanical ventilation, and aggressive physical cooling measures (Gillman, 1999). It is important to note that hyperthermia associated with SS does not result from hypothalamic thermoregulatory dysfunction but rather from excessive heat production due to sustained neuromuscular activity. Consequently, conventional antipyretic agents such as acetaminophen are generally ineffective and are not recommended as primary treatment (Poian and Chiavegatto, 2023). Instead, priority should be given to physical cooling methods, including cooling blankets and ice packs (Wali, 2025). When neuromuscular blockade is required, non-depolarizing agents such as vecuronium are preferred to avoid hyperkalemia and rhabdomyolysis-related arrhythmias associated with succinylcholine use (Wali, 2025).

For patients with frequent or persistent seizures, benzodiazepines such as lorazepam or diazepam are recommended for symptom control (Gillman, 1999). These agents enhance γ-aminobutyric acid (GABA)–mediated neurotransmission and provide sedative, anxiolytic, and anticonvulsant effects, thereby alleviating agitation, myoclonus, and muscle rigidity while helping stabilize vital signs (ZHANG et al., 2025). In contrast, although propranolol may reduce blood pressure through partial antagonism of 5-HT1A receptors, its effectiveness in controlling the core manifestations of SS is limited because the primary pathophysiological mechanism involves 5-HT2A receptor–mediated neuromuscular hyperactivity; therefore, it is not routinely recommended (Nisijima et al., 2001). Some experimental evidence also suggests that tianeptine may mitigate potential serotonergic drug-induced neurotoxicity in brain tissue (Ates et al., 2025).

## 4 Conclusion

This case highlights that SS may occur even though the overall incidence associated with concomitant tramadol and linezolid use is considered low in real-world population-based studies. These data may not fully reflect the risk in vulnerable subgroups. In the present case, severe SS developed rapidly in an elderly patient with multiple comorbidities and polypharmacy, underscoring the importance of individualized risk assessment in clinical practice.

Therefore, when linezolid is used in combination with serotonergic agents such as tramadol, early recognition of neuromuscular and autonomic symptoms and prompt discontinuation of the suspected drugs should be considered essential. Rapid symptom improvement within hours after drug withdrawal in this case further supports timely cessation as a key determinant of favorable outcome.

## Data Availability

The original contributions presented in the study are included in the article/supplementary material, further inquiries can be directed to the corresponding author.
